# Impulsivity and sexual addiction: factor structure and criterion-related validity of the sexual addiction screening test in Mexican adults

**DOI:** 10.3389/fpsyt.2023.1265822

**Published:** 2023-11-21

**Authors:** Diana Mejía Cruz, Laurent Avila Chauvet, Luis Villalobos-Gallegos, Christian Gabriel Toledo-Lozano

**Affiliations:** ^1^Psychology Department, Sonora Institute of Technology, Obregon City, Mexico; ^2^School of Medicine and Psychology, Autonomous University of Baja California, Tijuana, Mexico; ^3^Research Coordination, National Medical Center “20 de Noviembre” ISSSTE, Mexico City, Mexico

**Keywords:** sex addiction, impulsivity, reward/punishment responsivity, personality, psychological distress

## Abstract

Sexual addiction is associated with serious health problems. Due to that fact, it is quite important to perform a comprehensive assessment. The Sex Addiction Screening Test (SAST-R) is a self-administered questionnaire with good psychometric properties used in several countries. Our study conducts a cross-cultural adaptation of the SAST-R on the Mexican population. The original version of the SAST-R was translated into Mexican Spanish, and we performed a pilot with 23 participants to be sure that the participants understood the meaning of the items. The final version was administered to 370 adults who completed the SAST-R, and measures of impulsivity (the Kirby questionnaire), reward/punishment responsivity (BIS-BAS scale), personality (BIG-Five), and psychological distress (SCL-90). The confirmatory factor analysis (CFA) with a five-factor model with one second-order factor model had the best fit. Reliability analysis suggests acceptable internal consistency (*α* = 0.80). The SAST-R scores exhibited significant correlations with several variables. Specifically, they showed a positive correlation with the neuroticism scale (*r* = 0.11, *p* < 0.05), a negative correlation with the conscientiousness scale (*r* = –0.21, *p* < 0.01), a negative correlation with the BIS scale (*r* = −0.11, *p* < 0.05), and a positive correlation with psychological distress (*r* = 0.34, *p* < 0.01). Notably, there were no significant correlations observed with variables that we initially expected to have a substantial association, such as impulsivity (*r* = –0.004, *p* > 0.05) and the three BAS subscales (*p* > 0.05). We found with an algorithm that psychological distress, impulsivity, neuroticism, and agreeableness were the good predictors to identify high scores of hypersexuality. Our results confirmed that the Mexican Spanish version of the SAST-R has good psychometric properties to be used in future research.

## Introduction

Excessive sexual behavior can represent a serious health concern. For individuals affected by this condition, the intensity of their sexual urges and behaviors is associated with clinical distress or impairment in social, occupational, or other areas of functioning (1). Individuals with sex addiction tend to engage in compulsive masturbation, affairs, intercourse with sex workers, pornography use, and cybersex, and often engage in sexual behavior in response to feelings of depression, anxiety, boredom, loneliness, or other negative affective states (2). According to Kafka (1), hypersexuality is characterized by the amount of time spent engaging in sexual obsessions; moreover, these individuals display significant impairment in the control of their sexual behavior characterized by disinhibition and impulsivity. The criteria for sex addiction or hypersexuality are quite similar to those proposed by Zuckerman (3) when defining addictive disorders: intense craving, compulsivity, lack of control, and continuation of the behavior even after adverse consequences.

Given the health problems caused by sex addiction and its common comorbidities such as substance use, anxiety, and mood disorders, it is essential to have tools for a comprehensive assessment. The Sex Addiction Screening Test (SAST-R) is one of the most widely used in different countries by its validity and reliability (4), and the instrument explores the experience of child sexual abuse and insecure attachment patterns that are considered risk factors for sexual addiction. The SAST-R is a 20-item self-administered questionnaire with dichotomous responses. It was revised in 2010 (SAST-R) and was studied in a sample of 1,604 individuals diagnosed with sexual addiction, and the results indicated that the single-factor model of measurement was the best fit. A cutoff score of 5 and 6 points (out of 20) is suggested for sexual addiction in heterosexual men and women, respectively (4, 5). Twenty-five items were added in the revised version, resulting in an instrument of 45 items, grouped into four subscales to conduct differential measurements: heterosexual men, women, and homosexuals, and internet use for sexual acts. The internal consistency was measured using Cronbach's alpha, depending on the group (heterosexual men, women, and homosexuals), ranging from 0.868 to 0.904. In this version of SAST-R (4), four factors were identified using exploratory principal components analysis (affect disturbance; relationship disturbance; preoccupation, and loss of control). The aforementioned four factors explain 44% of the variance in results. Despite these changes, the authors maintain a five-factor scale categorization, which includes affect disturbance, relationship disturbance, loss of control, preoccupation, and associated features such as experiences of childhood sexual abuse, sexual problems of parents, or involvement in sexual activities with minors.

The revised version of the SAST-R was translated and validated among Polish individuals (5). The results of this validation process confirmed its five-factor structure, which accounted for 63.64% of the variance in measures of sexual addiction. The Cronbach's alpha coefficient was found to be α = 0.904, and the cutoff value was based on five test points. In contrast, other versions based on the first version of SAST (6), a French adaptation (7) and a Spanish adaptation (8) of the SAST-R with 25 items, found different factor structures. In the Spanish adaptation (8), the authors found a four-factor structure that explained 57.39% of the variance and reliability ranging from 0.82 to 0.85; due to these psychometric properties, and the cultural differences in language, we decided to use the revised version of SAST-R (4) using only 20 items. Another significant rationale for selecting the 20-item version was its inclusion of the full spectrum of sexual orientations. This inclusion renders the test more comprehensive in its ability to detect problems related to sex addiction on a shorter scale. Furthermore, these additional 25 items displayed lower correlations with the dimensions of preoccupation, loss of control, relationship disturbance, and affect disturbance. This version includes heterosexual men, women, and homosexuals (4). The French adaptation (7) found a one-factor structure that explained 31% of the variance, internal consistency KR-20 = 0.90, and the cutoff value on 13 test points. In all cultural adaptations of the SAST-R, the scale has been related to other measures of sexual disturbance; however, its relationship to other important concepts such as impulsivity, personality, reward/punishment responsivity, and psychological distress has been less explored. Sex addiction is associated with excessive time consumed on sexual behaviors, interfering with other important activities and obligations, engagement in response to dysphoric mood states and in response to stressful life events, unsuccessful efforts to control sexual urges, and the risk for physical, and emotional harm to self, and others (1, 9).

A previous research study has indicated that specific personality traits are linked to sex addiction. These traits include lower levels of agreeableness, conscientiousness, and openness, along with higher levels of neuroticism. Additionally, individuals with a propensity for seeking sensations, as assessed through Big Five and BIS-BAS (Behavioral Inhibition System/Behavioral Activation System) tests, have exhibited a greater likelihood of experiencing sex addiction (10–12).

Moreover, investigations into the relationship between impulsivity and hypersexuality have yielded notable insights. Positive correlations between sex addiction and various measures of impulsivity have been documented (11, 13–15). These findings underscore the interplay between excitatory and inhibitory mechanisms in the context of sex addiction, shedding light on the complex nature of this phenomenon.

Given the existing literature on personality traits, impulsivity, and their potential associations with sex addiction, it is crucial to rigorously evaluate the criterion validity of the SAST-R as a diagnostic tool. This endeavor holds promise for enhancing our understanding of the underlying mechanisms and factors contributing to sex addiction, which, in turn, can inform prevention and intervention strategies.

In order to improve comprehension of the underlying processes involved in the development of sex addiction, it is important to evaluate the correlation between the SAST-R and other relevant constructs. The aim of this study was to analyze the factor structure and criterion-related validity of the 20-item version of the SAST-R in the Mexican population. As the external criteria for SAST-R validity, we will use self-report measures of impulsivity (Kirby's Questionnaire), reward/punishment responsivity (BIS-BAS test), personality (Big-Five), and psychological distress (SCL-90). Based on previous research, we expected to find a high positive correlation between impulsivity, reward sensitivity, extroversion, openness, psychological distress, and SAST-R scores. Additionally, we predicted negative correlations between punishment sensitivity, conscientiousness, agreeableness, neuroticism, and SAST-R scores.

Finally, we developed a CART model to predict whether individuals could be classified as hypersexual or not, utilizing the cutoff defined in the Polish adaptation (5). The CART algorithm was employed to analyze the interactions among factors associated with hypersexuality. We explored the proportion of explanation by considering constructs related to impulsivity, reward/punishment responsivity, personality, and psychological distress. The CART algorithm simplifies the graphical interpretation of conditional relationships among various factors. Furthermore, it facilitates the handling of outliers or non-normalized data.

## Methods

### Design

We performed a cross-sectional design and used snowball sampling. The sample was recruited through online social media platforms. Participants were contacted through online advertisement and invited to participate in the study. All the participants were from urban areas of Mexico, aged between 18 and 50 years old, and with skills to write and read. All the participants filled out all the questionnaires to be part of the current sample.

### Participants

We included 370 adult participants, of which 301 participants were women (*age*: *M* = 26.8, *SD* = 6.59), 62 participants were men (*age*: *M* = 26.4, *SD* = 6.02), five participants belonged to non-binary gender (*age*: *M* = 23.4, *SD* = 4.15), and two (*age*: *M* = 20.5, *SD* = 2.12) participants did not report their gender. The average of education years was *M* = 17.06 (*SD* = 4.04), and the monthly income was *M* = 370.68 USD (*SD* = 628.39). The sample consisted of participants who were married (11.4%), divorced (1.4%), single (74.3%), concubinage (9.5%), and dating someone [not seriously; (3%)]. The participants did not exhibit any psychiatric diagnoses, neurological disorders, or substance use disorders. This information was obtained through their responses to three initial questions following informed consent.

The study was approved by the Sonora Institute of Technology Institutional Review Board (ID 178) approved the protocol. Additionally, participants provided written informed consent to comply with the Declaration of Helsinki. Participants did not receive economical compensation for their participation. Participants acknowledged that they were unaware of the study hypotheses.

### Measures

A set of structured online surveys was used to collect information regarding demographics (i.e., age, education in years, gender, income, neurological diagnoses, and psychiatric diagnoses). To minimize the risk of participant withdrawal from the study, we included 236 items, distributed in 33 pages with an expected completion time of 20–30 min. Measures included those as follows:

#### Sexual addiction

The recent version of the Sexual Addiction Screening Test-Revised comprises 20 basic test items. We chose the 20-item version because the items are considered for heterosexual men, heterosexual women, homosexual men, and women. This version of the scale measures sexual addiction into five categories, described as follows:

(1) Affect disturbance (A), a significant decrease in mood, with high levels of anxiety and depression associated to own sexual behaviors and their consequences (items 4, 5, 11, 13, and 14); (2) Relationship disturbance (R), one's own sexual behavior is related to significant struggles in close relationships (items 6, 8, and 16); (3) preoccupation (P), obsessive thoughts about sexual behaviors (items 3, 18, 19, and 20); (4) loss of control (C), individuals are unable to manage sexual behaviors despite the problems and their consequent costs (items 10, 12, 15, and 17); (5) associated features (F), four questions that cover experiencing sexual abuse during childhood, perception of sexual problems of parents, and undertaking sexual activities with under aged persons (items 1, 2, 7, and 9). According to Carnes et al., the first four factors describe crucial symptoms of addiction (4), and traumatic sexual experiences are features associated with compulsive sexual behaviors. We compute each subscale as units of analysis.

#### Impulsivity (Kirby's questionnaire)

Kirby's (16) Monetary Choice Questionnaire is a measure of impulsivity that assesses an individual's tendency to choose immediate, smaller rewards over delayed, larger rewards. The 27 questions of the Kirby questionnaire are divided into three sets of 9 each, based on whether the delayed reward is small ($25, $30, or $35), medium ($50, $55, or $60), or large ($75, $80, or $85). The *k* values were calculated based on the hyperbola function: $V=\frac{A}{\left(1+kX\right)}$, where V is the subjective value of the delayed outcome, *k* is a parameter reflecting the discount rate at which the subjective value decreases as the delay until receiving the outcome increases, and X is the delay. The higher values of *k* represent more impulsivity.

#### Big five personality model

The 60-item NEO-FFI is a measure of the Big Five personality traits, with well-established reliability and validity (17). This measure consists of 240 items 5-point Likert scale that ranges from 'strongly disagree' to 'strongly agree.' The instrument has the following subscales: extroversion (e.g., full of energy and generates a lot of enthusiasm), neuroticism (e.g., can be tense and worries a lot), conscientiousness (e.g., does things efficiently, makes plans, and follows through with them), agreeableness (e.g., has a forgiving nature and likes to cooperate with others), and openness (e.g., is ingenious, a deep thinker, is curious about many different things). It has been established as an efficient and reliable measure (rtt = 0.80) with Spanish Speaking Latino groups (18, 19). We compute each subscale as units of analysis.

#### Behavioral activation/inhibition

The BIS/BAS scale consists of twenty statements with a Likert scale of four options, ranging from “totally agree” to “strongly disagree.” The items are grouped into two large subscales: behavioral inhibition system (BIS), with seven items, and behavioral activation system (BAS), with thirteen items. The BAS subscale is divided into fun seeking (four items), reward responsivity (five items), and drive (four items). We compute each subscale as units of analysis. The value of Cronbach's alpha is 0.853, and the scale is well correlated with substance abuse in the Mexican population (20).

#### Co-occurring psychiatric symptoms

Symptom Checklist 90 (SCL-90): through 90 items on a Likert scale, from 0 to 4. It evaluates the degree of psychological distress experienced by the subject during the period between the day of evolution and 1 week prior to application (21). The value of Cronbach's alpha of internal consistency for seven of all nine dimensions, as well as the ISG, was >0.7. At the same time, the rest obtained scores higher than 0.66. We used it for analysis of the total score of the instrument.

### General procedures

The translation and adaptation to the original version of the SAST-R for the Mexican context were conducted by two clinical psychologists. The translated version was then reviewed by two experts in addiction psychology, both with Master's degrees, who provided feedback that was incorporated into the final version. We followed the retroversion procedure according to test translation guidelines with a native speaker (22). We performed a pilot study with 23 participants to be sure that the participants understood the meaning of the items. In the pilot phase, participants reported a strong comprehension of all 20 items. Consequently, we retained the version to preserve its integrity.

The anonymous survey was designed using Google Forms with encryption and sent to participants. During data collection, the participants did not provide any personal information such as names or last names. Participants were enrolled over 18 years old. An ID number was assigned to participants to guarantee their anonymity. All the data was stored in a secured server to which only the principal researcher had access. Before starting, informed consent was shown to the participants. The survey started if they agreed to participate; otherwise, no information was collected. The recruited sample was living in several urban areas of different states in Mexico.

The data collection (i.e., behavioral battery tests and demographic information) was carried out between October 2021 and May 2022. All participants used the same website. After participants agreed to join the study, the participants started the behavioral test battery in the following order: SAST-R, Kirby questionnaire, Big Five, BIS-BAS, and SCL-90. The participants took, on average, approximately 30 min to respond to all the surveys.

### Data analysis

This study used a cross-sectional design. We calculated means, standard deviations, and frequencies for all sample demographic characteristics to determine the appropriate statistical test for correlations and intergroup comparisons. A normality analysis was conducted, including the Kolmogorov–Smirnov test and Shapiro–Wilk test. Additionally, Levene's test was used to evaluate the homogeneity of variances for each dependent variable. The results of these tests indicated that non-parametric tests would be more appropriate for the data. We considered G^*^Power software latest version 3.1.9.7 for the sample size with α = 0.05, β = 0.8, and five predictors for a lineal multiple regression. The current sample size (*n* = 370) is according to the G^*^Power analysis.

For the construct validity, we fitted the five-factor model of SAST-R (4, 5) using confirmatory factor analysis (CFA). Items were considered ordinal variables; therefore, we used weighted least squares with mean and variance adjusted (WLSMV) estimator. The fit of the CFA was tested through root mean square error of approximation (RMSEA), χ^2^, comparative fit index (CFI), and Tucker–Lewis's index (TLI) (23, 24). The following cutoff was considered scores: RMSEA scores below 0.05 indicate a good fit, whereas scores between 0.05 and 0.08 are an acceptable fit; TLI score >0.95 indicates a good fit; and a CFI score of > 0.95 indicates an excellent fit (25). Regarding χ^2^, a good fit is indicated when χ^2^/df ≤ 2, whereas χ^2^/df ≤ 3 is indicative of an acceptable fit (26). CFA was performed using Mplus 8.6. We fitted four models: the original five-factor models as specified by Carnes et al. (4), and Gola et al. (5), and their respective second-order models with five first-order factors (the same as the original models, but each factor loaded in a second-order factor). It is crucial to emphasize that the model proposed by Carnes et al. (4) comprises 27 items categorized into six factors, which include the five original factors and five additional items about internet use for sexual acts, whereas Gola's model incorporates only 20 items distributed across five factors (5). Consequently, these models do not exhibit nesting properties. Given this non-nested nature, we exclusively examined the comparison between the original and high-order factors, employing the DIFFTEST option in Mplus 8.6. This was the sole viable approach for assessing non-nested models when employing the WLSMV estimation method.

Whenever the χ^2^ test yielded statistical significance at a significance level of a *p* < 0.05, it indicated that the high-order factor model exhibited a superior fit. Subsequently, we selected the model with the most favorable overall fit indices for further analysis. Additionally, we conducted a Pearson's *r* between the individual latent score and the sum score to determine the most appropriate. Cronbach's α was performed to assess internal consistency.

For the criterion-related validity analysis, we computed Pearson correlations and regression model among the SAST-R total score and the score in the scales: *k* value (impulsivity); total score of extroversion scale; total score of neuroticism scale; total score of conscientiousness scale; total score of agreeableness scale; total score of openness scale; total score of BIS scale; total score of fun-seeking scale; total score of reward responsivity; total score of drive scale; and total score of SCL-90 scale.

To contrast hypersexuality and no-hypersexuality in each variable (impulsivity, Big-Five factor personality, reward/punishment responsivity, and psychological distress). We established a categorical variable, differentiating between scores equal to or lower than five and scores exceeding five in the SAT-R test. Our selection of these values closely aligned with those of the original version (4). The Mann–Whitney *U*-test was performed to compare the differences between hypersexuality and no-hypersexuality groups for the performance in the dependent variables.

To evaluate the prediction of the SAST-R test scores, we employed the Classification Regression Tree (CART) algorithm and logistic regression analysis. The algorithm was trained using 33% of the data and tested on 67%. The decision tree was constructed with a maximum of three splits and no cross-validation was used. Each node has decision rules, the probability of cases, the Gini index, and the number of cases. The lower Gini indexes suggest higher information gain or uncertainty reduction [see details in Mejía et al. (27)]. The predicted group is found in the leaves of each last node. The statistical analyses were performed using Python 3.11 including Sklearn libraries.

## Results

### Construct validity

**Figure 1:**
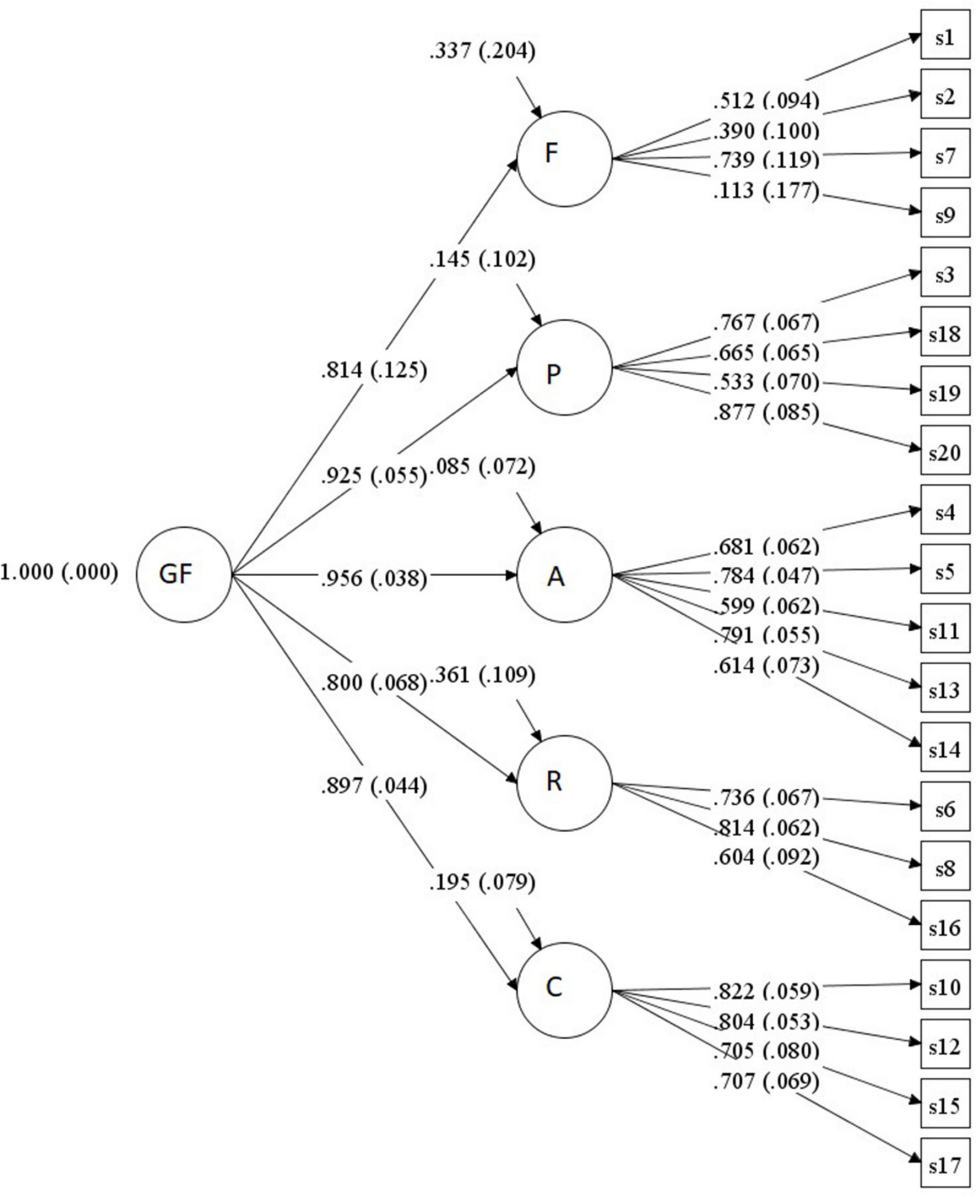
Results of confirmatory factor analysis. F, Associated features; P, Preoccupation; A, AFFECT disturbance; R, Relationship disturbance; C, Loss of control.

According to the theoretical assumptions of Carnes et al. (28) and Gola et al. (5) about a five-factor model, we used a confirmatory factor analysis to verify the hypothesis of a five-factor model with and without the one second-order factor model. The fit of the five-factor model with one second-order factor model based on Gola et al. (5) proved to be the most adequate (see Table 1 and Figure 1). We performed Pearson correlation between the latent score, as calculated by the CFA and the total score, and we obtained *r* = 0.96; therefore, further analysis will be performed using the sum scores.

**Table 1:** Fit measures of SAST-R factor models.

	**Models**			
	**a**	**b**	**c**	**d**
χ^2^ test (df)	438.942 (309)^*^	460.153 (318)^*^	254.401 (160)^*^	266.000 (165)^*^
CFI	0.928	0.921	0.935	0.930
TLI	0.918	0.913	0.922	0.919
RMSEA	0.034	0.035	0.040	0.041
RMSEA 90% C.I.	0.026–0.041	0.028–0.042	0.030–0.049	0.031–0.049
SRMR	0.133	0.137	0.119	0.122

a, five-factor model from Carnes et al. (28); b, five-factor with one-second-order factor model based on Carnes et al. (28); c, five-factor model from Gola et al. (5); d, five-factor with the one-second-order factor model based on Gola et al. (5). Model b had a better fit than Model a (χ^2^[9] = 27.602, *p* = 0.0011); Model d had significantly better fit than Model c (χ^2^[5] = 14.913, *p* = 0.0107). ^*^p < 0.05.

### Reliability

Cronbach's alpha coefficient was 0.80 for the scale, which indicated that the SAST-R scale was reliable. In contrast, the reliability for each scale of the five-factor model indicated that factors three (affect disturbance) and five (loss of control) have α > 0.60, and the other factors (relationship disturbance, preoccupation, and associated features) have a low α of < 0.50.

### Criterion-related validity

In the correlation matrix, we found significant correlations among SAT-R with the variables: neuroticism scale (*r* = 0.11, *p* < 0.05), conscientiousness scale (*r* = –0.21, *p* < 0.01), BIS scale (*r* = –0.11, *p* < 0.05), and psychological distress (*r* = 0.34, *p* < 0.01). These correlations were constant in four of the five factors. The factor of relation disturbance was correlated with the agreeableness scale (*r* = – 0.11, *p* < 0.05) and the openness scale (*r* = 0.10, *p* < 0.05 (see Table 2).

**Table 2:** Correlations among the SAST-R, impulsivity, psychological distress, personality traits, and reward/punishment responsivity.

**Variables**	**SAST-GF**		**SAST-F**		**SAST-P**		**SAST-A**		**SAST-R**		**SAST-C**		**K**		**SCL-90**		**Extra**		**Cons**		**Agree**		**Neuro**		**Open**		**BIS**		**BAS-D**		**BAS-F**		**BAS-RR_ **
1. SAST-GF	—																																
2. SAST-F	0.963	^***^	—																														
3. SAST-P	0.988	^***^	0.945	^***^	—																												
4. SAST-A	0.995	^***^	0.953	^***^	0.976	^***^	—																										
5. SAST-R	0.936	^***^	0.902	^***^	0.918	^***^	0.920	^***^	—																								
6. SAST-C	0.977	^***^	0.926	^***^	0.957	^***^	0.965	^***^	0.892	^***^	—																						
7. K	−0.004		0.008		−0.005		−0.001		−0.034		−0.003		—																				
8. SCL-90	0.340	^***^	0.328	^***^	0.345	^***^	0.338	^***^	0.310	^***^	0.325	^***^	−0.014		—																		
9. Extra	−0.061		−0.021		−0.064		−0.072		−0.003		−0.069		0.017		−0.240	^***^	—																
10. Cons	−0.216	^***^	−0.185	^***^	−0.213	^***^	0.217	^***^	−0.192	^***^	−0.219	^***^	−0.044		−0.286	^***^	0.322	^***^	—														
11. Agree	−0.083		−0.082		−0.068		−0.093		−0.115	^*^	−0.057		−0.030		−0.133	^*^	0.193	^***^	0.312	^***^	—												
12. Neuro	0.111	^*^	0.123	^*^	0.111	^*^	0.110	^*^	0.116	^*^	0.094		−0.080		0.566	^***^	−0.204	^***^	−0.357	^***^	−0.267	^***^	—										
13. Open	0.078		0.097		0.066		0.077		0.103	^*^	0.070		−0.059		0.038		0.327	^***^	0.269	^***^	0.207	^***^	−0.029		—								
14. BIS	−0.119	^*^	−0.116	^*^	−0.117	^*^	−0.130	^*^	−0.101		−0.094		0.089		−0.347	^***^	0.203	^***^	0.277	^***^	0.117	^*^	−0.426	^***^	0.031		—						
15. BAS-D	0.084		0.064		0.096		0.086		0.038		0.086		−0.030		0.035		−0.169	^**^	−0.160	^**^	−0.051		0.083		−0.213	^***^	0.042		—				
16. BAS-F	−0.071		−0.094		0.075		−0.066		−0.093		−0.050		0.051		−0.063		−0.057		0.074		−0.047		0.028		−0.066		0.202	^***^	0.471	^***^	—		
17. BAS-RR	−0.069		−0.082		−0.071		−0.071		−0.099		−0.037		0.069		−0.052		−0.072		−0.082		−0.073		0.026		−0.050		0.184	^***^	0.434	^***^	0.553	^***^	—

K, K value; SCL 90, Symptom Checklist; Extra, Extraversion; Cons, Conscientiousness; Agree, Agreeableness; Neuro, Neuroticism; Open, Openness; BIS, Behavioral Inhibition Scale; BAS-D, Behavioral Activation Scale-Drive; BAS-F, Behavioral Activation Scale-Fun Seeking; BAS-RR, Behavioral Activation Scale-Reward Responsiveness; SAST-GF: General Factor; SAST-F – Associated features; SAST-P – Preoccupation; SAST-A – Affect Disturbance; SAST-R – Relationship Disturbance; SAST-C – Loss of control.

* *p* < 0.05,

** *p* < 0.01,

*** *p* < 0.001.

**Figure 2:**
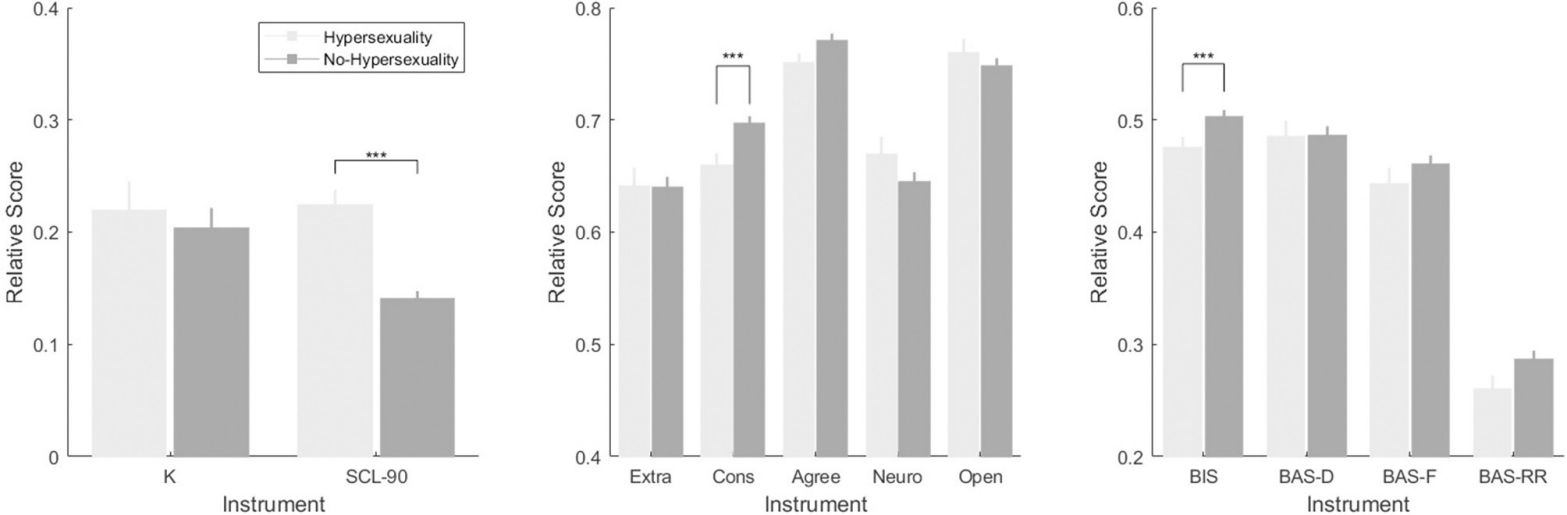
Hypersexuality and no-hypersexuality instrument scores. K, *K* value; SCL 90, Symptom checklist; Extra, Extraversion; Cons, Conscientiousness; Agree, Agreeableness; Neuro, Neuroticism; Open, Openness; BIS, Behavioral Inhibition Scale; BAS-D, Behavioral Activation Scale-Drive; BAS-F, Behavioral Activation Scale-Fun Seeking; BAS-RR, Behavioral Activation Scale-Reward Responsiveness. ^***^Statistical significance between hypersexuality, and no-hypersexuality in that variables.

With the categorical variable of hypersexuality and no-hypersexuality, we found significant differences between the groups in the variables: conscientiousness (*U* = 10902.5, *p* = 0.003, *r* = 0.197), BIS (*U* = 11354.5, *p* = 0.015, *r* = 0.164), and psychological distress (*U* = 18744.0, *p* = 0.001, *r* = 0.380). Marginal differences in the variables: agreeableness (*U* = 11829.0, *p* = 0.055, *r* = 0.129) and *k* value (*U* = 15235.5, *p* = 0.071, *r* = 0.122). The results show that individuals in the high hypersexuality group exhibited higher levels of impulsivity and psychological distress as well as lower levels of conscientiousness and agreeableness. Additionally, this group was found to have a lower Behavioral Inhibition System (see Figure 2).

**Figure 3:**
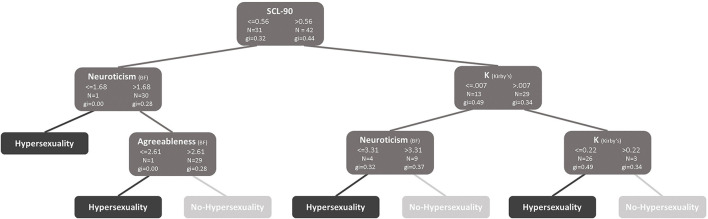
Classification and regression trees (CART) algorithm. K, *K* value; SCL 90, Symptom checklist.

In the logistic regression and Classification and Regression Trees (CART) algorithm, we introduced the variables for the criteria validity of SAST-R. We found in the first node that psychological distress (100% of the sample) is the best predictor of hypersexuality. In the second node, high impulsivity (57.1%) and low neuroticism (42.9%) identify cases of hypersexuality. In contrast, less psychological distress in the first node and high neuroticism (41.2%) in the second node identify no-hypersexuality. In the second node, high impulsivity (*k* value, 39.4%) and less agreeableness (41.2%) identify hypersexuality (See Figure 3).

## Discussion

Our study aimed to investigate the factor structure, internal consistency, and criterion-related validity of the SAST-R. An additional objective was to develop a CART model to predict whether individuals were classified as hypersexual or not hypersexual. We found that the SAST-R had good reliability in the general factor; however, the five subscales of the model had lower values of reliability. This finding is consistent with the previous adaptation studies of the SAST-R (5, 7, 29). We consider that the SAST-R has a better interpretation as a single construct, which is supported by our findings, indicating that five first-order factors with one second-order factor model had a better fit. Additionally, the results of the fit measures are quite similar to previous studies using CFA (4, 5). These findings are consistent with the idea that sexual addiction is a single construct as proposed by Carnes et al. (28). In this regard, further research is warranted to identify the effect of the specific dimensions in the clinical presentation, course, severity of impairment, and comorbidities of sexual addiction, as previous studies suggest (30). It is important to note that we sampled the general population; therefore, assessing the clinical utility of the SAST-R dimension scores is outside our current scope. On the other hand, SAST-R, which has a strong correlation with the latent score of the high-factor model, could be used as a screening tool. This is supported by the positive correlation found between the SAST-R total score and other relevant measures.

In addition to the initial analysis, we conducted an exploratory factor analysis to compare its findings with those of the confirmatory analysis. In this exploratory analysis, we observed that the Kaiser–Meyer–Olkin (KMO) measure produced a value of KMO = 0.830, indicating the appropriateness of the dataset for factor analysis. Furthermore, Bartlett's test of sphericity yielded statistical significance (χ^2^ = 1312.637, *p* < 0.001), providing further evidence of the suitability of the intercorrelations among variables for the execution of factor analysis.

A total of six factors were extracted from the dataset, collectively explaining 54.58% of the total variance. However, the analysis revealed that changes in the loading of five items (items: 3, 9, 11, 16, and 19) led to their association with different factors, thereby altering the subscales related to associated factors, preoccupation, affect disturbance, and relationship disturbance. Consequently, we believe that conducting a confirmatory analysis that considers the associations with evidence in the context of sex addiction would yield more robust results.

Our results demonstrate criterion-related validity for the SAST-R. Significant correlations were found between the SAST-R scores and psychological distress, impulsivity, the behavioral inhibition system, and personality traits such as neuroticism, conscientiousness, and agreeableness. These correlations align with previous research that has found associations between hypersexuality, dysphoric mood states, difficulty in controlling sexual urges, and potential harm to oneself and others (1, 9). These findings serve to mitigate the impact of the low internal consistency observed within each of the five subscales.

In the current study, we found that psychological distress is the best predictor of high scores of hypersexuality. A potential limitation is that the sample size resulted in insufficient power to detect the differences between individuals classified as hypersexual against no-hypersexual. In addition, personality traits such as less agreeableness and neuroticism provide information on the vulnerability to hypersexuality, which is related to relationship disturbance, affect disturbance, and preoccupation in previous studies (10–12, 28).

In terms of loss of control, our findings support previous research that has identified a positive association between impulsivity and hypersexuality (11, 13–15). Specifically, we found that individuals who scored higher on impulsivity measures, such as preferring immediate small rewards over delayed larger rewards also scored higher on measures of hypersexuality. This is consistent with the idea that impulsivity plays a significant role in hypersexuality and loss of control, rather than being part of an obsessive-compulsive disorder model (31, 32).

With the impulsivity measure, we provide information about how to consider hypersexuality as an addiction disorder following criteria: compulsion, loss of control, and continuing the behavior despite experiencing adverse consequences (3). Although previous studies suggest the view of hypersexuality as a behavioral addiction, currently, the data are insufficient to completely support this conclusion, such as previous meta-analysis (33, 34). Based on our findings, we recommend conducting further research to explore the underlying cognitive processes associated with hypersexuality, using cognitive tasks such as the Iowa gambling task to measure risk-taking, the Columbia card task to measure decision-making and tasks that assess the affective and deliberative processes. These tasks would provide a more detailed understanding of how cognitive processes contribute to developing and maintaining hypersexuality.

A limitation of our study is that the scores of our sample were relatively lower than those from clinical samples; therefore, it is possible that the extrapolation of the results for the CART might be limited in settings with a higher prevalence of individuals with compulsive sexual behavior. Therefore, we suggest in future studies to evaluate clinical samples, which was hard in the current study due to the lack of treatment institutions where we can find the participants in the north of the country. Moreover, we strongly recommend exploring comorbidities and other potential factors associated with sex addiction, such as substance abuse. In the evaluation process, we suggest incorporating a battery of assessments to measure cognitive impairment, hypomanic episodes, and substance abuse.

Another limitation of our study is that we had relatively fewer male participants, and the proportion of the male-to-female ratio of participation in online surveys is relatively constant (one man for three women); however, it is important to increase the sample of males in Latino population in future studies to prevent a possible misrepresentation in the results and explore the gender differences such as previous studies (35).

Given the limited availability of tools for measuring hypersexuality and sexual dysfunction in Latino populations, our findings suggest that the Mexican version of the SAST-R is useful for further research on hypersexuality in this population. We found that the validity and internal consistency of the test are comparable to previous studies, and it is a reliable and valid instrument for assessing hypersexuality in Mexican individuals.

Our results confirmed that the Spanish version of the SAST-R had evidence to support its psychometric properties in the Mexican population, allowing the measure can be used in future research. Finally, we may conclude that many factors affect sex addiction, such as psychological distress, personality traits, low behavioral inhibition system, and high impulsivity.

## Data availability statement

The raw data supporting the conclusions of this article will be made available by the authors, without undue reservation. Requests to access these datasets should be directed to diana.mejia175562@potros.itson.edu.mx.

## Ethics statement

The studies involving humans were approved by the Sonora Institute of Technology Institutional Review Board (ID 178) approved the protocol. The studies were conducted in accordance with the local legislation and institutional requirements. The participants provided their written informed consent to participate in this study.

## Author contributions

DM: Conceptualization, Data curation, Formal analysis, Funding acquisition, Investigation, Methodology, Project administration, Resources, Supervision, Validation, Visualization, Writing—original draft, Writing—review & editing. LA: Data curation, Formal analysis, Investigation, Software, Writing—review & editing. LV-G: Data curation, Formal analysis, Investigation, Methodology, Supervision, Writing—review & editing. CT-L: Funding acquisition, Methodology, Project administration, Resources, Supervision, Writing—review & editing.
